# A phase IB study of ipilimumab with peginterferon alfa-2b in patients with unresectable melanoma

**DOI:** 10.1186/s40425-016-0194-1

**Published:** 2016-12-20

**Authors:** Andrew S. Brohl, Nikhil I. Khushalani, Zeynep Eroglu, Joseph Markowitz, Ram Thapa, Y. Ann Chen, Ragini Kudchadkar, Jeffrey S. Weber

**Affiliations:** 1 Department of Cutaneous Oncology, H. Lee Moffitt Cancer Center and Research Institute, 12902 Magnolia Drive, Tampa, FL 33612-9416 USA; 2 Department of Biostatistics and Bioinformatics, H. Lee Moffitt Cancer Center and Research Institute, Tampa, FL USA; 3 Winship Cancer Institute of Emory University, Atlanta, GA USA; 4 New York University Langone Medical Center, New York, NY USA

**Keywords:** Melanoma, Ipilimumab, Peginterferon alfa-2b, Immunotherapy, Clinical trial

## Abstract

**Background:**

Ipilimumab and peginterferon alfa-2b are established systemic treatment options for melanoma that have distinct mechanisms of action. Given the need for improved therapies for advanced melanoma, we conducted an open-label, single institution, phase Ib study to assess the safety and tolerability of using these two agents in combination.

**Methods:**

Study treatment consisted of ipilimumab given every 3 weeks, for a total of four infusions, concurrent with peginterferon alfa-2b administered subcutaneous weekly for a total of 12 weeks. This was followed by maintenance therapy with peginterferon alfa-2b administered subcutaneously weekly for up to 144 additional weeks. The study was designed as a two-stage dose escalation scheme with continuous dose-limiting toxicity monitoring during the induction phase.

**Results:**

Thirty one patients received at least 1 dose of study treatment and 30 were assessable for efficacy endpoints. We found that ipilimumab at 3 mg/kg dosing with peginterfeon alfa-2b at 2 μg/kg/week was the maximum tolerated dose of this combination. The incidence of grade 3 drug-related adverse events (AEs) was 45.2%. There were no grade 4/5 AEs. The overall response rate was 40% by immune-related response criteria. Median progression-free survival was 5.9 months. The median overall survival was not reached with at a median follow-up of 35.8 months.

**Conclusions:**

We report that the combination of ipilimumab at 3 mg/kg dosing combined with peginterfeon alfa-2b at 2 μg/kg/week demonstrated an acceptable toxicity profile and a promising efficacy signal. Further study of this combination is warranted.

**Trial registration:**

ClinicalTrials.gov identifier: NCT01496807, Registered December 19th, 2011.

## Background

Melanoma is amongst the most rapidly increasing cancers, with over 76,000 new invasive cases predicted in the United States in 2016 [1]. Over recent years, treatment for metastatic melanoma has significantly evolved with the development and regulatory approval of several therapeutic classes of medications including both molecularly targeted and immunologically focused checkpoint inhibitor therapies. Despite these recent advances, limitations remain in the treatment of advanced melanoma. Treatment with molecularly targeted therapies, while initially highly effective for the majority of patients whose tumors harbor a *BRAF* V600 mutation, typically lead to development of treatment resistance [2–4]. While offering the potential for durable responses, treatment with checkpoint inhibitor immunotherapies remain active in only a percentage of patients with advanced melanoma, and most patients will require further therapy [5–7]. Combination checkpoint inhibitor therapy is associated with high rates of response but is also limited by severe toxicity in more than half of treated patients [8].

Interferon alfa has long been an adjuvant treatment option for patients with high-risk resected melanoma since its FDA approval in 1995. An important observation from adjuvant trials utilizing inferferon treatment is that dose and duration of therapy may be an important determinant of survival [9, 10]. Peginterferon alfa-2b was developed to facilitate increased exposure to the interferon alfa 2b molecule as compared to the nonpegylated version. Adjuvant use of peginterferon alfa-2b was approved by the US FDA in 2011 for stage III melanoma after showing an improvement in relapse-free survival compared to observation a in phase III study [11].

Ipilimumab, a monoclonal antibody directed against the cytotoxic T-lymphocyte antigen 4 (CTLA-4), was the first checkpoint inhibitor approved for use in melanoma after showing efficacy in two phase III trials [12, 13]. Though treatment with antibodies directed against the programmed death 1 (PD-1) protein have largely supplanted ipilimumab as first line therapy for advanced melanoma, ipilimumab continues to have an important therapeutic role either as part of combination checkpoint inhibitor therapy or as a second line immunotherapy option for those patients with progression after PD-1 inhibitor therapy.

Given the need for improved efficacy of available systemic therapies for advanced melanoma, we conducted a phase IB study of the combination of ipilimumab and peginterferon alfa-2b in patients with unresectable melanoma. These agents were selected based on the proven efficacy of either of these two agents alone as well as the non-overlapping mechanism of action. Interferon alfa treatment has been shown to induce an inflammatory tumor microenvironment, with upregulation of major histocompatibility complex antigen processing and co-stimulatory molecules and induction of T helper type 1 (Th1) polarization [14–19]. The antitumor impact of interferon, however, may be suppressed by tumor immune tolerance mechanisms, leading to limited clinical activity of interferon as a single agent. CTLA-4 is a key regulator of immune tolerance and a negative regulator of T cell activation [20, 21]. We therefore hypothesized that CTLA-4 inhibition might alter the balance of tumor tolerogenic mechanisms and potentiate the clinical activity of the interferon-induced inflammatory microenvironment. In addition to the immunologic rationale of this combination, we were motivated by the encouraging results of a phase II study of treatment with high-dose interferon alfa-2b plus the anti-CTLA-4 antibody tremelimumab showed acceptable toxicity and a response rate of over 30% [22]. We hypothesized that the addition to peginterferon alfa-2b to ipilimumab treatment might enhance the efficacy of CTLA-4 blockade. The primary objective of the study was to assess the safety and tolerability of this combination. The secondary objectives were to determine response rates by immune-related response criteria (irRC), progression free survival, and overall survival. In patients treated with either interferon alfa-2b or ipilimumab, retrospective analyses have suggested that there is an association of autoimmune phenomena with clinical benefit [23–26]. Therefore an additional planned exploratory endpoint was to assess the rate of autoimmune antibody induction and whether this was associated with anti-tumor response with the combination.

## Methods

This was a phase Ib, open-label study conducted at the H. Lee Moffitt Cancer Center (NCT01496807) under an IND exemption grated by the FDA. The main inclusion criteria were: patients age ≥16 years with a histological and/or cytological confirmed diagnosis of unresectable stage IIIB/C-IV melanoma untreated systemically other than a BRAF inhibitor for metastatic disease; adequate renal, hepatic, and hematologic parameters; and ECOG performance status of 0 or 1. Patients with a history of severe cardiac comorbidities, uncontrolled diabetes or thyroid dysfunction, history of HIV seropositivity, patients suffering from active autoimmune disease other than controlled hypothyroidism or vitiligo, pre-existing severe psychiatric conditions, patients on systemic corticosteroid therapy for any reason, and patients with uncontrolled brain metastases were excluded. The study was approved by the institutional review board at the H. Lee Moffitt Cancer Center in Tampa, FL. All patients provided written informed consent.

Study treatment consisted of ipilimumab given every 3 weeks, for a total of four infusions, concurrent with peginterferon alfa-2b administered subcutaneous weekly for a total of 12 weeks. This was followed by maintenance therapy with peginterferon alfa-2b only, administered subcutaneously weekly for up to 144 additional weeks. The study was designed with a two-stage dose escalation scheme with continuous dose-limiting toxicity (DLT) monitoring during the induction phase of the first dose level (DL) and a total enrollment of 36 planned to observe 30 evaluable patients. The first cohort of up to 15 patients would be given ipilimumab at a 3 mg/kg dose and peginterferon alfa-2b at 3 μg/kg/week. Dose escalation to DL 2 (ipilimumab at 10 mg/kg) would follow if all 15 patients in DL 1 had initiated treatment, and fewer than 3 of 10 patients have had a DLT after completing 12 weeks of treatment. If the number of DLTs during the ipilimumab induction period in either cohort exceeded ≥2 in first 6 patients, ≥3 of the first 10 patients, or ≥5 of the first 15 patients, and the DLT was not felt to be an immune-related adverse event (irAE) caused by ipilimumab, then patients thereafter would be treated at DL -1 with ipilimumab 3 mg/kg week and peginterferon 2 μg/kg/week until a total of 30 patients would be treated. At the reduced dose of peginterferon (DL -1), again continuous DLT monitoring was performed during the induction phase and a second dose reduction in peginterferon (DL -2) could be undertaken if excessive peginterferon alfa-2b related DLTs were observed. The dose escalation plan of the study is summarized in Table 1.

**Table 1:** Dose escalation study design

Dose Level	Peginterferon alfa-2b	Ipilimumab
−2	1 μg/kg/week	3 mg/kg
−1	2 μg/kg/week	3 mg/kg
1	3 μg/kg/week	3 mg/kg
2	3 μg/kg/week	10 mg/kg

Dose modification guidelines for peginterferon alfa-2b were pre-specified and similar to the package insert for this drug. Similar to the EORTC 18991 study, the intent of the protocol was for the investigator to modify the dose of peginterferon alfa-2b as needed in order to keep the patient on treatment while maintaining a ECOG performance status of 0 or 1. Dose reduction of ipilimumab was not allowed. Guidelines for ipilimumab dose skipping, discontinuation, and management of ipililimumab-related irAEs were prespecified and similar to the package insert.

Safety assessments were performed at during induction therapy and every 3 months during the maintenance phase of the trial by history and physical examination, ECOG performance status evaluation, and regular monitoring of hematology, blood chemistry, and urinalysis. Thyroid function was monitored on screening and at week 12 and months 6, 12, 15, 18, 21, 24, 27, 30, 33, and 36. Additionally, presence of vitiligo as an autoimmune event was assessed with a Wood’s Lamp at each clinic visit. Beginning at the week 12 visit, disease status was monitored by physical examination and imaging studies every 12 weeks during years 1–3 until disease progression or the treatment regimen was concluded. For patients who came off treatment for toxicity, they were monitored for PFS with CT scans every 3 months up to year 3, then every 6 months thereafter indefinitely. Brain MRI for disease monitoring was completed at every other imaging evaluation. Once a patient progressed, they were followed for OS every 3 months indefinitely.

Blood samples for autoimmune antibody assessments were collected pre-treatment immediately prior to the first dose and at week 6, 12 and then every 3 months up to month 36. Samples were taken immediately prior to ipilimumab and/or peginterferon alfa-2b administration and were tested by enzyme-linked immunosorbent assays (Quanta lie, Inova Diagnostics) for antinuclear antibodies, anti-DNA antibodies, antithyroglobulin antibodies, antimicrosomal antibodies, and anticardiolipin antibodies. Samples from month 15 on were stored and would only be assessed if equivocal data arise from the samples taken during the first 12 months of therapy.

## Results

Between February 17th 2012 and December 5th 2013, 33 patients were screened and 31 patients were enrolled on treatment. The median patient age was 65 [range 38–83]. There were 24 cutaneous primaries, 5 unknown primaries and 2 acral melanomas. All patients had stage IV disease at the time of trial enrollment. Most patients were systemic therapy naïve. Approximately 80% of patients had a normal baseline serum lactate dehydrogenase (LDH) level. A significant majority of patients had tumors that were *BRAF* V600 wild type. Additional baseline characteristics of the treatment population are detailed in Table 2.

**Table 2:** Baseline characteristics of the treatment population (*n* = 31) at the time of study enrollment

Baseline Characterisitcs of Treatment Population (*n* = 31)	
Median age (range)	65 (38–83)
Gender, *n* (%)	
Male	18 (58.1%)
Female	13 (41.9%)
ECOG performance status	
0	11 (35.5%)
1	20 (64.5%)
Primary site, *n* (%)	
Cutaneous	24 (77.4%)
Unknown	5 (16.1%)
Acral	2 (6.5%)
M substage, *n* (%)	
M1a	3 (9.7%)
M1b	9 (29.0%)
M1c	19 (61.3%)
LDH level, *n* (%)	
Normal	25 (80.6%)
Elevated	6 (19.4%)
BRAF V600, *n* (%)	
wild type	26 (83.9%)
V600E	3 (9.7%)
V600K	2 (6.5%)
Prior systemic therapy, *n* (%)	
None	28 (90.3%)
BRAF targeted therapy	1 (3.2%)
cytotoxic chemotherapy	1 (3.2%)
adjuvant vaccine clinical trial	1 (3.2%)

Seven patients were treated at Dose Level (DL) 1, and the remaining 24 at DL-1. Overall, 27 patients (87%) completed the 3 month induction phase of treatment. Three patients discontinued prior to this first planned restaging due to early disease progression, and 1 was taken off study prior to completing 1 cycle due to a change in diagnosis. Ten additional patients had progression of disease at the time of first restaging and so did not proceed with the maintenance phase of the trial. Of the 17 remaining patients that completed the induction phase of treatment without disease progression, only 1 completed the full 3 years of treatment, including the 144 additional weeks of maintenance peginterferon alfa-2b. Eight patients discontinued maintenance treatment due to later disease progression and the remaining 8 due to toxicity and/or patient preference.

### Dose limiting toxicity and maximum tolerated dose

The first seven patients initiated treatment on DL1, and 3 patients experienced G3 gastrointestinal symptoms (nausea, vomiting and/or abdominal pain), which was considered to be a DLT related to peginterferon alfa-2b. As per pre-specified criteria, subsequent patients were treated at DL -1, with peginterferon alfa-2b reduced from 3 μg/kg/week to 2 μg/kg/week. At DL -1, pre-specified DLT limits were not reached in the first 15 patients treated at this dose, establishing this dosing schedule as the maximum tolerated dose (MTD) and allowing for further cohort expansion at this dose level to complete enrollment.

### Safety

The safety population was comprised of all 31 patients receiving at least 1 dose of study drug. All patients experienced grade 1 toxicity, 28 patients (90.3%) experienced at least grade 2 toxicity, and 14 patients (45.2%) experienced a grade 3 toxicity felt by investigators to be attributable to treatment (Table 3). Eight patients (25.8%) experienced a grade 3 immune related adverse event felt related to ipilimumab treatment including rash (4), colitis (2) and endocrinopathies (2). All were managed with steroids with resolution or hormone replacement, as appropriate. Ten patients (32.2%) experienced a grade 3 toxicity related to peginterferon alfa-2b therapy, most commonly hematologic (4) and gastrointestinal (3). All peginterferon alfa-2b related toxicities resolved with supportive care and treatment interruption/discontinuation. Neither any grade 4 toxicities nor treatment-related deaths occurred.

**Table 3:** Drug related adverse events in the safety population (*n* = 31), including any AE experienced by ≥5% of the cohort and all grade 3 AEs. Note that there were no grade 4 or 5 drug related AEs

Toxicity category	Description	Grade 1–2	Percent	Grade 3	Percent
Blood and lymphatic system disorders	Anemia	5	16.1%	-	-
Cardiac disorders	Atrial fibrillation	-	-	1	3.2%
Endocrine disorders	Hypophysitis	1	3.2%	1	3.2%
	Hypothyroidism	2	6.5%	1	3.2%
Eye disorders	Dry Eye	3	9.7%	-	-
Gastrointestinal disorders	Abdominal Pain	2	6.5%	-	-
	Colitis	2	6.5%	2	6.5%
	Constipation	4	12.9%	-	-
	Diarrhea	17	54.8%	-	-
	Dry Mouth	5	16.1%	-	-
	Flatulence	2	6.5%	-	-
	Nausea	16	51.6%	2	6.5%
	Vomiting	8	25.8%	1	3.2%
General disorders and administration site conditions	Chills	19	61.3%	-	-
	Fatigue	28	90.3%	1	3.2%
	Fever	15	48.4%	-	-
	Flu like symptoms	7	22.6%	-	-
	Injection site reaction	7	22.6%	-	-
	Infusion reaction	3	9.7%	-	-
	Malaise	3	9.7%	-	-
	Night Sweats	4	12.9%	-	-
Investigations	Alanine aminotransferase increased	8	25.8%	-	-
	Alkaline phosphatase increased	2	6.5%	-	-
	Aspartate aminotransferase increased	8	25.8%	-	-
	Lymphocyte count decreased	4	12.9%	1	3.2%
	Neutrophil count decreased	3	9.7%	3	9.7%
	Platelet count decreased	6	19.4%	-	-
	Weight loss	5	16.1%	-	-
	White blood cell decreased	8	25.8%	2	6.5%
Metabolism and nutrition disorders	Anorexia	16	51.6%	-	-
	Dehydration	1	3.2%	2	6.5%
	Hyponatremia	-	-	2	6.5%
Muskuloskeletal and connective tissue disorders	Arthralgia	15	48.4%	1	3.2%
	Myalgia	2	6.5%	-	-
Nervous system disorders	Dizziness	5	16.1%	-	-
	Dysgeusia	8	25.8%	-	-
	Headache	17	54.8%	-	-
	Syncope	-	-	1	3.2%
Psychiatric disorders	Anxiety	3	9.7%	-	-
	Depression	5	16.1%	-	-
Respiratory, thoracic and mediastinal disorders	Cough	7	22.6%	-	-
	Dyspnea	8	25.8%	-	-
	Productive Cough	2	6.5%	-	-
Skin and subcutaneous tissue disorders	Alopecia	6	19.4%	-	-
	Dry Skin	4	12.9%	-	-
	Pruritus	18	58.1%	2	6.5%
	Rash	22	71.0%	4	12.9%
	Skin hypopigmentation	7	22.6%	-	-
Any		31	100%	15	48.4%

### Efficacy

Thirty patients were evaluable for efficacy. One additional patient experienced rapid disease progression shortly after initiation of therapy and underwent surgery for spinal cord compression. Pathological evaluation of this specimen revealed that the patient was affected by a high-grade carcinoma, rather than a melanoma as was believed based on previous biopsy. This patient was taken off further study treatment and was not considered evaluable for efficacy endpoints.

**Fig. 1:**
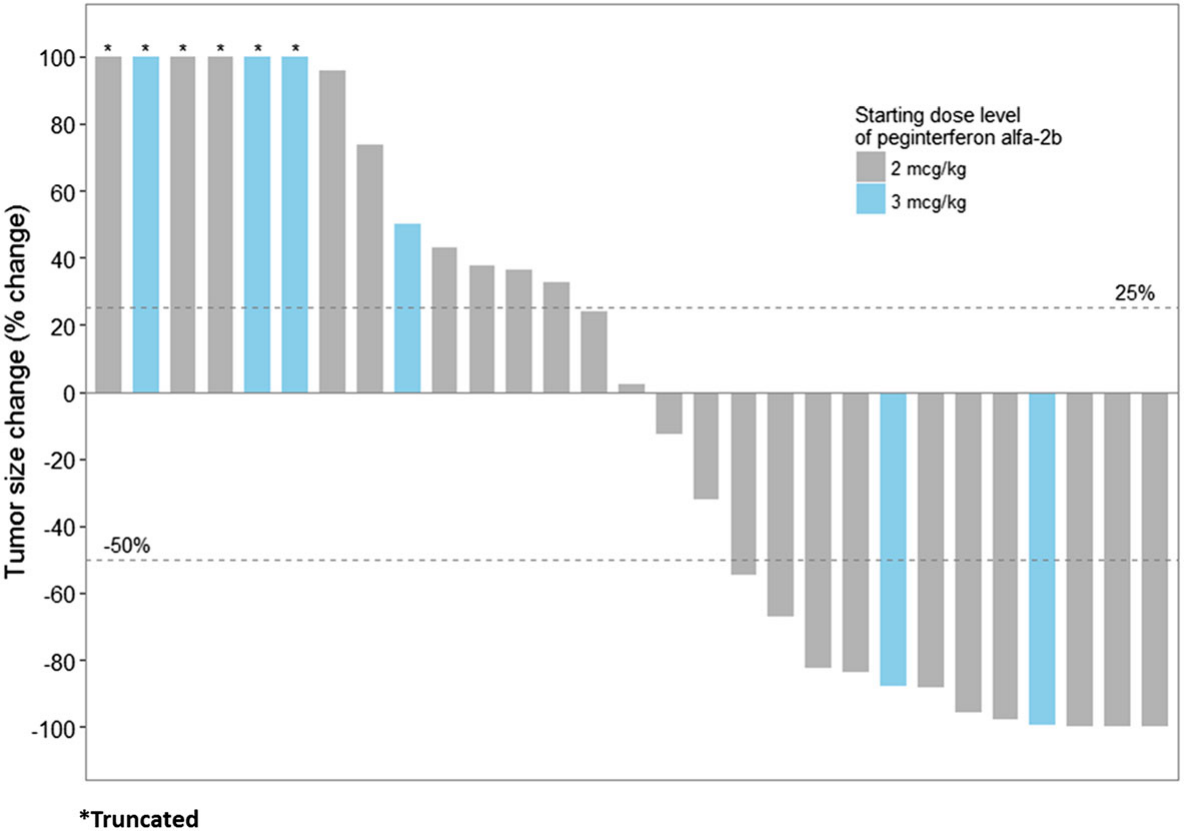
Waterfall plot depicting best response as a percentage change from baseline of target lesions. irRC cutoffs for partial response and progressive disease are shown (*dotted lines*)

**Fig. 2:**
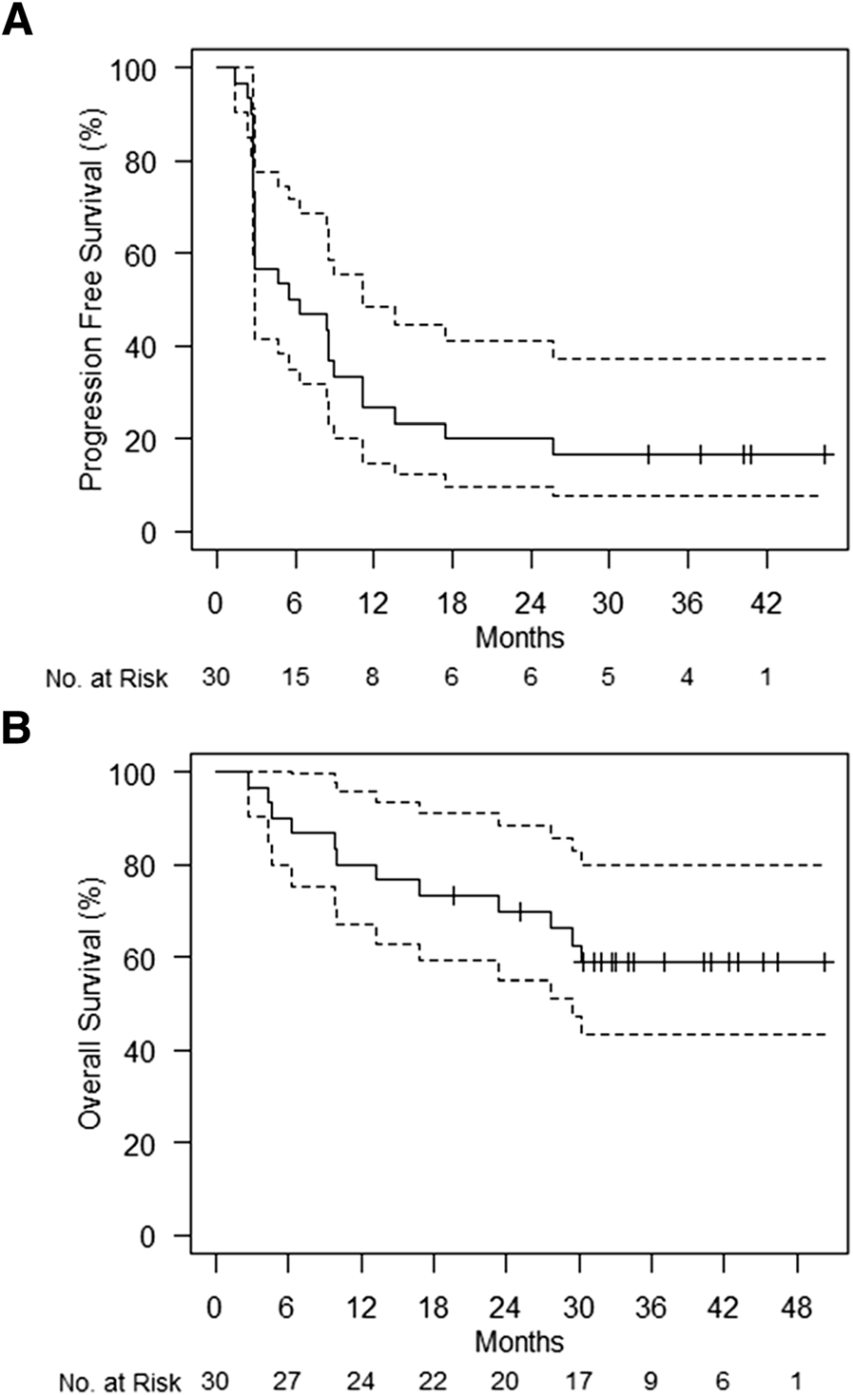
Kaplan-Meier plots of progression free survival (**a**) and overall survival (**b**) with 95% confidence intervals

Of 30 evaluable patients, 12 (40%) experienced a confirmed response (1 CR, 11 PR) by irRC. Three additional patients had stable disease of at least 24 weeks (SD), for a disease control rate (CR+PR+SD) of 50% (Fig. 1). There was no statistical difference in the efficacy rates between DL1 and DL-1, though the study was not powered to detect a difference. The median progression free survival was 5.9 months (Fig. 2a). Five patients (16.7%) remain alive and progression free without further treatment with a median follow-up of 40.3 months (range 33.0–46.4 months). The median overall survival was not reached in this study, with 18 patients (60%) alive at the time of censoring with a median follow-up of 35.8 months (range 19.7–50.2 months) (Fig. 2b).

### Autoimmunity as a biomarker of treatment response

Seven patients were diagnosed with autoimmune vitiligo during the study period. Notably, 6 of these patients (85.6%) experienced an objective response, and the remaining patient experienced stable disease of >1 year. The correlation between vitiligo and objective response was highly statistically significant (*p* = 0.009). The median time from start of treatment to development of vitiligo was 224 days (range 140–374). In an attempt to mitigate lead-time bias of the association of vitiligo with positive clinical outcome, we performed landmark analysis of overall survival between those with and without vitiligo at 6, 9, and 12 month time points. Overall survival of patients presenting with vitiligo was not significantly improved compared with patients alive but free of vitiligo at these time points (*p* = 0.94, *p* = 0.46, *p* = 0.69, respectively).

Four of the patients had a positive screening result for an autoimmune antibody at the time of study entry. Twelve patients developed a positive result during treatment for one or more antibodies of a previously negative screen, including antithyroglobulin (7), anti-DNA (5), anticardiolipin IgM (2), anticardiolipin IgG (2) and antinuclear (1). There was no significant association between development of a new positive autoantibody and objective disease response (33.3% with a new autoantibody positive vs. 44.4% without, *p* = 0.71).

## Discussion

We report the final results of clinical trial NCT01496807, an open label, phase IB study of ipilimumab with peginterferon alfa-2b in patients with unresectable stage IV melanoma. We found that ipilimumab at 3 mg/kg plus peginterferon alfa-2b at 2 μg/kg weekly was the maximal tolerated dose of this combination. Though not the primary endpoint of this study, we noted a promising efficacy signal with 40% of patients experiencing an objective response, 17% of patients having prolonged progression free survival without further therapy, and the majority of patients remaining alive with approximately 3 years of follow-up data. We also note that the development of vitiligo as an autoimmune toxicity was highly associated with disease response, consistent with previous reports [27, 28]. Survival of patients developing vitiligo was not significantly improved versus to those who did not experience this toxicity when compared using landmark analysis to mitigate lead-time bias. These comparisons, however, were limited by small patient numbers.

The significant toxicities observed on this study were well within the range of those expected based on the individual toxicity profiles of the two agents used. In the EORTC 18991 study, peginterferon alfa-2b use was associated with a 57% rate of G3 toxicity at the 3 μg/kg/week dose [29]. We found this dose combined with ipilimumab at 3 mg/kg to have an unacceptably high rate of G3 toxicity, requiring hospitalizations in 3 of the first 7 patients treated. Within the context of combination therapy this rate of early hospitalizations due to peginterferon could potentially compromise the ability to receive adequate checkpoint inhibition. The type of ipilimumab-related immune related adverse events was also very similar to previous reports, with rash, colitis and endocrinopathies being observed. It should be noted, however, that the rate of G3 ipilimumab-related toxicities, and in particular G3 rash (13%), was higher than in most previous reports of single agent ipilimumab use at this dose [25]. Though it is possible that ipilimumab-related immune toxicities were potentiated by the addition of peginterferon, we speculate that the enhanced efficacy observed with this combination, leading to greater numbers of patients surviving long enough to experience delayed toxicities, may also contribute.

The efficacy observed in this trial appears superior to previous reports of single agent ipilimumab use [13, 30]. Though this study was not designed for assessment of efficacy endpoints, the response rate observed is promising when considering ipilimumab monotherapy response rates in prior trials. One previous phase II study of treatment with interferon alfa plus anti-CTLA-4 also noted encouraging efficacy [22]. Differences in baseline characteristics and response criteria between studies limit direct comparison of these two studies. Notably, patients in our study were predominantly treatment naïve and with normal baseline LDH levels, both of which may be associated with more favorable outcome. Despite differences in study design and agents used, the favorable efficacy signal in both studies collectively supports the idea of future investigation of similar combination therapy approaches. We caution, however, that our study results need to be viewed in context of recent advances in the treatment of advanced melanoma. Specifically, this trial was conducted prior to availability of PD-1 inhibitor therapy, and all patients on trial were checkpoint inhibitor therapy naïve. As ipilimumab monotherapy is now more commonly given in the setting of PD-1 inhibitor failure, future study of interferon alfa combined with checkpoint inhibition will likely focus on PD-1 inhibitor combinations, for which studies are already ongoing, or in the second line setting with ipilimumab after failure of PD-1 inhibitor based therapy. Our study also does not address the optimal duration of peginterferon alfa-2b maintenance therapy, or whether a maintenance phase is even beneficial. Of note, only a single patient in this study completed the planned 3 year peginterferon maintenance.

## Conclusions

Despite recent advances in the treatment of advanced melanoma, there is a need for continued improvement in treatment options. We report that the combination of ipilimumab at 3 mg/kg dosing combined with peginterfeon alfa-2b at 2 μg/kg/week demonstrated an acceptable toxicity profile and a promising efficacy signal. Further study of this combination is warranted. More generally, this trial adds to the growing rationale that novel combinations of immunotherapeutic agents should be investigated in advanced melanoma.

## Abbreviations

AE: Adverse event
CTLA-4: Cytotoxic T-lymphocyte antigen 4
DL: Dose level
DLT: Dose-limiting toxicity
irAE: Immune-related adverse event
irRC: Immune-related response criteria
LDH: Lactate dehydrogenase
MTD: Maximum tolerated dose
PD-1: Programmed death 1
